# Impact of Socio-Demographic Factors, Financial Burden, and Social Support on Anxiety and Depression Symptoms in Puerto Rican Women with Breast Cancer

**DOI:** 10.3390/bs15070915

**Published:** 2025-07-05

**Authors:** Paulette Ayala-Rodríguez, Dayaneira Rivera-Alers, Manuel Rivera-Vélez, Jovanny Díaz-Rodríguez, Mercedes Ramirez-Ruiz, Carolina Quiles-Bengochea, Cristina I. Peña-Vargas, Zindie Rodriguez-Castro, Cynthia Cortes-Castro, Guillermo N. Armaiz-Pena, Eida M. Castro-Figueroa

**Affiliations:** 1 School of Medicine, Ponce Health Sciences University, Ponce, PR 00716, USA; payala23@stu.psm.edu (P.A.-R.); garmaiz@psm.edu (G.N.A.-P.); 2 Ponce Research Institute, Ponce, PR 00716, USA; dayrivera@psm.edu (D.R.-A.); manuelrivera@psm.edu (M.R.-V.); jovadiaz@psm.edu (J.D.-R.); mramirez@psm.edu (M.R.-R.); cquiles@psm.edu (C.Q.-B.); cpena@psm.edu (C.I.P.-V.); zrodriguez@psm.edu (Z.R.-C.); ccortes@psm.edu (C.C.-C.); 3 Public Health Program, Ponce Health Sciences University, Ponce, PR 00716, USA; 4 School of Behavioral and Brain Sciences, Ponce Health Sciences University, Ponce, PR 00716, USA; 5 School of Dental Medicine, Ponce Health Sciences University, Ponce, PR 00716, USA

**Keywords:** social determinants, financial burden, social support, breast cancer, Hispanic, Puerto Rico, psycho-oncology, anxiety, depression, mental health

## Abstract

Breast cancer (BC) is the leading cancer diagnosis among women in Puerto Rico. Psychological distress is prevalent in this population, and social determinants may exacerbate this risk. This study examines whether sociodemographic characteristics, financial burden, and social support levels are associated with symptoms of anxiety and depression in Puerto Rican women with BC. A quantitative secondary analysis was conducted on a sample of 208 Hispanic women with BC, utilizing the Patient Health Questionnaire (PHQ-8) and the Generalized Anxiety Disorder (GAD-7) questionnaire. These scores were compared with sociodemographic values and Interpersonal Support Evaluation List (ISEL-12) scores, establishing statistical significance through association, parametric, and non-parametric tests, and regression models. 38.5% and 26.4% of participants showed clinically significant symptoms of depression and anxiety, respectively. Age and perceived income showed significant associations with psychological outcomes. However, regression analysis revealed perceived income as the only significant predictor for both depression and anxiety. Tangible and belonging support were significantly lower in participants with symptoms of depression, while appraisal support was significantly lower in participants with symptoms of anxiety. Findings highlight the influence of perceived financial stress on mental health and the need for psychosocial interventions tailored to the patients’ economic context.

## 1. Introduction

### 1.1. Background and Relevance

According to the Puerto Rico Central Cancer Registry, breast cancer is the most prevalent type of cancer among women on the island (2016–2020), accounting for 30.5% of all cancers diagnosed in women. It is also the most common cause of cancer-related deaths, accounting for 18.6% of all deaths in women with cancer in Puerto Rico (PR). This means that roughly 2319 women receive a breast cancer diagnosis each year, while 432 women die annually from the disease. It is estimated that one in nine women will be diagnosed with breast cancer at some point in their lives (Torres-Cintrón et al., 2023). This raises significant concerns, as breast cancer impacts many women and the annual incidence rates continue to rise (NCI, n.d.a).

86.8% of women with breast cancer in PR will still be alive five years after their initial diagnosis (Torres-Cintrón et al., 2023). Survival rates, however, vary with the stage of the disease at the time of diagnosis (NCI, n.d.a). According to the National Cancer Institute’s SEER statistics, women are most often diagnosed with breast cancer between the ages of 65 and 74 (27.4%), with a median diagnosis age of 63 years. Breast cancer death rates continue to decrease each year, while the 5-year survival rates keep improving (ACS, 2024; NCI, n.d.a). These statistics are encouraging and can be attributed to a deeper understanding of the disease over the years, along with improved adherence to screening and therapeutic methods. Early screening also aids in detecting the disease at earlier stages, providing women with a better chance of survival.

Breast cancer patients are at a higher risk of developing mental health problems following their diagnosis (Breidenbach et al., 2022; Fradelos et al., 2017; Tsaras et al., 2018). Their new reality forces them to worry about many things, such as suffering and death, leaving their loved ones, changes in appearance, and not fulfilling their life plans or obligations. Financial toxicity, a term used to describe problems that arise in cancer patients related to the costs of medical care, their health insurance, or other financial concerns regarding their health care, is also among these concerns (Hamel et al., 2017; NCI, n.d.b). Furthermore, this toxicity can be increased by the possibility of losing a job and the inability to cover living expenses, which can generate financial stress in this population and affect their quality of life (Khazi et al., 2023; Knight et al., 2018). A possible strategy to mitigate this problem is discussions between the patient and their oncologist about the costs associated with treatment by incorporating economic considerations into the clinical decision-making process (Hamel et al., 2017).

Receiving a diagnosis can be a traumatic experience, often resulting in symptoms of distress and anxiety (Fortin et al., 2021). This process begins before receiving the diagnosis, marked by the uncertainty of waiting for cancer confirmation when an abnormality is detected. It can then continue with informing family and friends, particularly if they have children (Breidenbach et al., 2022; de Sousa Barros et al., 2018; Khazi et al., 2023). Having a family history of cancer can also influence how the patient feels going forward (Khazi et al., 2023), as well as their expectations regarding treatment and survival. When incidence rates of cancer are high among family members, comparing their experiences in terms of disease process and outcomes can lead to assumptions, for example, that their own cancer could end in death. This perception can affect aspects such as mental health (Liu & Cao, 2014).

After beginning treatment, symptoms of distress and anxiety may decrease as the patient adjusts to their new reality. However, the prevalence of depressive symptoms increases during this period, and they may also develop post-traumatic stress disorder (PTSD) symptoms like intrusion and avoidance (Brown et al., 2020; Caceres et al., 2022; Knight et al., 2018). PTSD is more likely to develop in younger women diagnosed with breast cancer (Wu et al., 2016). Another contributing factor is having a history of stress and trauma (de Sousa Barros et al., 2018).

Anxiety and depression levels are higher in women with a more advanced or metastatic cancer. The cancer stage is also linked to pain and other debilitating symptoms (Caceres et al., 2022; Tsaras et al., 2018; Lueboonthavatchai, 2007). Additionally, social determinants can influence the risk and persistence of psychological symptoms during the recovery process, affecting some women more than others based on their past experiences, lifestyle, and environment. Studies have shown that social determinants such as place of residence, marital status, religious affiliation, physical activity, and education level are linked to the presence of anxiety and depression in patients with breast cancer. Tsaras et al. (2018) demonstrated that residing in rural areas can heighten the risk of both anxiety and depression. This increased risk may stem from restricted access to care and lower income (Islami et al., 2024).

According to the U.S. Census Bureau, around 39.6% of the population in PR lives below the poverty level. The average annual household income is $25,621, with most Puerto Rican households (21.1%) earning less than $10,000 (U.S. Census Bureau, 2023a, 2023b; Vargas-Ramos et al., 2023). Furthermore, as shown by the Economic Development Bank for PR, the economy of PR has been declining since 2005, with the most recent recessions occurring in 2017, 2019, and 2022. These recessions are largely due to the natural disasters that have impacted the island, which are becoming more frequent and stronger every year (Rodriguez-Rabassa et al., 2020). A low income can hinder patients from affording and accessing the medical services they require, including medications, doctor visits, tests, and mental health care (Knight et al., 2018). The lack of access to mental health care, either because of the inability to afford it or to recognize the need for help, further prevents patients from receiving the care they need.

However, most of Puerto Rico’s population benefits from government health coverage (Reforma), Medicare, or private insurance. Coverage and access to services can vary significantly. First, the government plan provides health care to residents with low incomes and is publicly funded. However, it is administered by private insurers, leading to a limited network of providers, and variable coverage and copayments for mental health services. Medicare, on the other hand, benefits the elderly population, as well as people with disabilities. It is important to note that it requires supplemental plans for comprehensive coverage. Private health insurance can offer more comprehensive care but is less accessible for those without the resources to afford it (Portela & Sommers, 2015).

Another significant aspect influenced by social determinants is cancer prevention. Uninsured women are less likely to receive a mammography test compared to those with private insurance or Medicaid (ACS, 2022; Islami et al., 2024). Education level is also linked to screening adherence. Women with lower education levels demonstrate a reduced likelihood of keeping their breast cancer screenings up to date or having undergone one within the past two years once they reach 45 years of age (ACS, 2022). While health insurance and education are certainly important, other social factors also play a big role in shaping health outcomes. Things like race, ethnicity, income level, and social marginalization can strongly affect whether someone gets screened for breast cancer. For instance, Black and Hispanic women are less likely to be diagnosed at an early stage than white women, pointing to ongoing gaps in access to screening and medical care. Systemic racism and long-standing social inequalities also make it harder for marginalized communities to get the screenings and follow-up care they need, which in turn contributes to higher breast cancer death rates in these groups (ACS, 2022).

These are not unique to the United States and Puerto Rico; international evidence reflects similar patterns. For example, a recent review conducted by Fenech and Gaffiero (2025) in Malta found that lower education was associated with a higher refusal rate for mammography screenings. Lower income correlated with decreased screening attendance, and widowed women were less likely to participate in breast cancer screening programs (Fenech & Gaffiero, 2025).

Considering these issues, which involve not only aspects related to an individual’s condition, such as breast cancer, but also how external factors play a crucial role in their health, proposals such as the biopsychosocial model of health and disease emerged as a mitigation strategy. This model, proposed by Engel (1977), developed the concept of improving the biomedical model by considering psychological and social aspects as part of an individual’s well-being and treatment of certain diseases (Bolton, 2023). This would not only put physical health into perspective but would also encompass mental health, which is often not prioritized in the same way.

### 1.2. Study Aims and Objectives

This study aims to assess whether there is an association between social determinants (e.g., sociodemographic characteristics, financial burden, and social support) and symptoms of anxiety and depression in women with breast cancer in PR. Additionally, this study explores the biopsychosocial model of health and disease, suggesting that disease involves not only the physical aspect (physiological deviations from normal biological function) but also the psychological and sociocultural aspects. These factors influence how individuals experience illness (Miles, 2013).

## 2. Materials and Methods

### 2.1. Study Design and Samples

This study is a quantitative secondary analysis of data originally collected from the cohort study “Biopsychosocial predictors of tumor-associated inflammation and progression” by Castro-Figueroa, E.M., Armaiz, G.N., and colleagues at Ponce Health Sciences University/Ponce Research Institute (PRI) in PR, and Jim, H. at Moffitt Cancer Center in Florida. The dataset includes baseline assessments from 208 Hispanic women aged 21 and older, living in 25 towns in the southern, western, and central regions of PR. These participants had been diagnosed with breast cancer, stages 0 to 4, and were actively receiving treatment at the time of recruitment. They were recruited through a non-probabilistic convenience sampling at the Admissions Department of the St. Luke’s Episcopal Hospital in Ponce, the Perea Hospital- Metro Pavia Health System in Mayaguez, and nearby medical offices of collaborating physicians in the southern region of Puerto Rico. After being reviewed for eligibility and obtaining their consent to participate in the study, the baseline assessment was administered at the recruitment site, at the study site in PRI, or at their residence by third- and fourth-year doctoral students in psychology, between the years 2018 and 2023. For more information about recruitment and other methods, please refer to Castro-Figueroa et al. (2021) and Peña-Vargas et al. (2025).

### 2.2. Assessment Instruments

As part of the consenting process, participants who consented to be in the study were also asked to retrieve clinical information from their medical records; this allowed disease, treatment, and appointment scheduling information to be collected from primary data sources. Self-reported questionnaires were administered to participants with breast cancer during a visit to the study site and at their homes to assess their symptoms of anxiety and depression over a two-week period. The questionnaires include the Patient Health Questionnaire (PHQ-8) Depression Scale and the Generalized Anxiety Disorder (GAD-7) Questionnaire. The PHQ-8 items assess eight of the nine symptom criteria for major depressive disorder, as defined in the Diagnostic and Statistical Manual of Mental Disorders (5th ed., DSM-V) (American Psychiatric Association, DSM-5 Task Force, 2013).

The PHQ-8 ranges were established as reported by Kroenke et al. (2009), where a score of 0–4 indicates minimal or non-significant symptoms, 5–9 indicates mild symptoms, 10–14 indicates moderate symptoms, 15–19 indicates moderately severe symptoms, and 20–24 indicates severe depression symptoms. A cutoff score of 10 or higher on this scale is considered a strong indicator of clinical depression (Kroenke et al., 2009). The GAD-7 questions address seven of the nine criteria in the DSM-V used to diagnose generalized anxiety disorder. The GAD-7 ranges were first reported by Spitzer et al. (2006), where 0–4 is considered minimal or non-significant, 5–9 is mild, 10–14 is moderate, and 15–21 is severe symptoms of anxiety. A score of 10 or higher on the GAD-7 is a strong indicator of a generalized anxiety disorder diagnosis (Spitzer et al., 2006). Both questionnaires, the PHQ-8 and the GAD-7, have good psychometric properties, with measurement invariance across sociodemographic factors like gender, age, education, and country, allowing for reliable score comparisons across different groups (Vilela-Estrada et al., 2025).

Social support was assessed using the Interpersonal Support Evaluation List (ISEL-12), from which we focused on the first eleven questions addressing tangible support, appraisal support, belonging support, and self-esteem (Cohen et al., 1985). The types of interpersonal support measured in this analysis were tangible support, appraisal support, belonging support, as well as overall social support.

Tangible support questions focus on the perceived presence of practical assistance with daily tasks or material resources. The questions related to this type of support are question #2 (“If I need assistance to fix something in my house or to repair my car, there is someone that can help me.”) and #9 (“If I go to the airport early in the morning, I would have difficulties in finding someone that could take me.”). The questions regarding belonging support, which assess whether the patient feels a sense of belonging, companionship, and social connection with others, are questions #5 (“When I feel alone, there are several people I could call or talk to.”) and #10 (“I feel that I am not always included in my friend circle.”). Appraisal support questions measure the emotional support, encouragement, and positive feedback participants receive. The questions addressing this type of support on the ISEL-12 are #1 (“There are several people that I trust to help solve my problems.”), #6 (“There is no one that I feel comfortable talking about intimate personal problems.”) and #11 (“There really is no one who can give me an objective view of how I’m handling my problems.”). The scores for the negative questions were adjusted accordingly. A higher score for tangible, belonging, and appraisal support, as well as social support, indicates a higher level of support. The ISEL-12 has an adequate internal consistency among languages and ancestry groups, validating its use in the Spanish-speaking Hispanic/Latino population (Merz et al., 2014).

In addition to the assessment instruments, sociodemographic and clinical variables were collected using self-administered questionnaires. These variables included age, marital status, cohabitation status, family history of cancer, diagnosis of depression, use of selective serotonin reuptake inhibitors (SSRIs), type of treatment received (chemotherapy, hormone therapy, radiation therapy, or surgery), presence of metastases annual household income, perception of economic adequacy, educational level, health insurance, and employment status. These variables were incorporated into statistical analyses to explore the existence of associations with symptoms of depression and anxiety, as described in the following section.

### 2.3. Statistical Analysis

The data was analyzed using IBM SPSS Statistics, version 29. The presence of significant anxiety and depression symptoms (scores of 10 or more on the GAD-7 and PHQ-8 questionnaires) were compared with levels of social support using Spearman’s correlation coefficient test and the Mann-Whitney U test, as well as with other demographic factors (age, income, marital status, living situation, household income, education level, and health insurance) using the Chi-square test. Binary logistic regression models were also performed to identify which factors are significantly associated with the presence of symptoms of depression (PHQ-8) and anxiety (GAD-7).

The Spearman’s correlation coefficient test measures the correlation between variables, indicating whether one variable increases (rho > 0) or decreases (rho < 0) as the other variable changes. This test was implemented to assess correlations between anxiety and depression symptoms (dependent variables) and social support (independent variables), given that the data do not follow a normal distribution. As the rho value gets closer to zero, the lower or less significant the correlation is between variables. The Chi-Square test determines if an association exists between two variables, the presence or absence of depression and anxiety symptoms (dependent variables) and the sociodemographic factors (independent variables), by measuring the difference between observed frequencies and expected frequencies. This test does not assume a causal relationship but rather evaluates the independence between variables. The difference between the mean scores of the first eleven questions of the ISEL-12 (independent variables) among participants with no depression or anxiety symptoms and those with depression or anxiety symptoms (dependent variables) was calculated using the Mann-Whitney U test. This nonparametric test was used to determine whether the populations significantly differ from one another, and to compare groups without assuming normality. Logistic regression analysis was performed to estimate the likelihood of depression and anxiety symptom outcomes, controlling for potential confounders (age, last academic level, marital status, income perception, annual income, employment, type of insurance, and depression). Results were expressed as adjusted odds ratios (ORs) with their 95% confidence intervals (95%CI) and *p*-values, with associations with a *p*-value < 0.05 considered statistically significant.

## 3. Results

### 3.1. Main Characteristics of the Study Sample

For this secondary analysis, data from 208 women diagnosed with breast cancer were examined. The average age of participants was 57 years, and most of them were married (44.2%). Those who were not married were either single (19.7%), widowed (14.4%), divorced (14.4%), or in a consensual union (7.2%) at the time of the study. A total of 51% lived with their spouse or partner, 23.1% lived with younger children, 24.5% lived with older offspring, and 15.4% lived alone (Table 1). Due to participants being allowed to select multiple options for living situation and type of treatment received, the sum of response frequencies surpasses the total sample size in these variables. Consequently, the sample size does not match the percentages shown in Table 1, as some participants chose multiple answers. The participants were treated by their oncologists based on their specific needs. Among the therapies utilized were chemotherapy (56.3%), radiotherapy (47.1%), hormone therapy (32.2%), and surgery (64.4%). 26 of these women had metastasis at the time of the original assessment (Table 1).

As shown in Table 1, the household income of most participants (41.2%) was below $12,000 per year. This aligns with the approximate rate of Puerto Ricans living below the poverty level (U.S. Census Bureau, 2023b). A total of 123 participants (59.7%) reported that their household income was insufficient to cover all expenses and needs, including rent, food, water, electricity, phone bills, medications, and more. For 37% of the women interviewed, education level was distributed as follows: no elementary school (1%), elementary school (2.9%), middle school (6.7%), and high school diploma (26.4%). The remaining 63% completed a higher education degree, such as a technical course or associate’s degree (22.6%), a bachelor’s degree (29.3%), or a master’s or doctorate degree (11.1%) (Table 1).

A total of 10.7% of participants had Medicare coverage, 33.7% had private health insurance, and 49.3% had government-issued health insurance known as “Reforma.” Only one participant (0.5%) reported not having any health insurance, and 4.9% reported having both private and government health insurance (Table 1). In terms of employment status, participants reported having a full-time job (25.5%), being unemployed (22.6%), or having already retired (24%). Additionally, there were participants who were disabled (10.6%), worked part-time (6.7%), were seasonal employees (1%), or held another employment status (9.6%), such as being a homemaker, not currently working due to cancer treatment, or self-employed (Table 1).

A cutoff score of 10 or higher was used in both the GAD-7 and the PHQ-8 to identify clinically significant symptoms of anxiety or depression. Among the 208 women who participated in the study, 40 (19.2%) reported clinically significant symptoms of depression, while 31 (14.9%) reported clinically significant symptoms of anxiety (Table 2).

### 3.2. Sociodemographic Factors

Participants over 65 years of age were less likely to exhibit symptoms of depression (OR = 0.378, 95% CI = 0.158–0.907, *p* = 0.025) and anxiety (OR = 0.179, 95% CI = 0.053–0.613, *p* = 0.002). Only 10.4% of participants in this age group experienced clinically significant symptoms of depression, while 4.5% reported clinically significant symptoms of anxiety (Table 3 and Table 4). Older participants were three times more likely to respond “yes” when asked if their household income is sufficient (OR = 3.093, 95% CI = 1.360–7.034); 62.9% of participants aged 65 or older indicated that they had enough income to meet all their needs and expenses (*p* = 0.006).

Participants under 50 were less likely to answer “yes” to the same question (OR = 0.411, 95% CI = 0.185–0.909), with 69.8% responding “no” (*p* = 0.026). A positive correlation was identified between participants aged 21–50 and appraisal support (rho = 0.230, *p* = 0.028), although this correlation was low. Conversely, a negative correlation was observed between participants aged 50–64 and tangible support (rho = −0.263, *p* = 0.011). Furthermore, they were twice as likely to exhibit symptoms of depression (OR = 2.036, 95% CI = 1.012–4.096, *p* = 0.044) (Table 3 and Table 4).

Table 3 and Table 4 also show that income is not associated with levels of anxiety and depression (*p* > 0.05). However, participants who reported that their income was sufficient were significantly less likely to have depression symptoms (OR = 0.382, 95% CI = 0.171–0.855). These participants were also less likely to have anxiety symptoms (OR = 0.366, 95% CI = 0.150–0.891). Among the women with sufficient income, 11% experienced symptoms of depression (*p* = 0.017), and 8.5% experienced symptoms of anxiety (*p* = 0.023) (Table 3 and Table 4).

Experiencing symptoms of depression or anxiety was significantly associated with a diagnosis of depression (*p* = 0.002 and *p* = 0.003, respectively) and with taking medication (*p* = 0.009 and *p* = ≤ 0.001, respectively). Among the women who reported suffering from depression, 45.8% exhibited symptoms of depression, and 37.5% exhibited symptoms of anxiety within the two weeks prior to their participation in the baseline assessment. This indicates that they are four times more likely to have depression symptoms (OR = 3.855, 95% CI = 1.560–9.524) and anxiety symptoms (OR = 3.900, 95% CI = 1.507–10.095). Of the women who reported taking SSRIs, 38.5% also had symptoms of depression as well as anxiety symptoms. Participants taking SSRIs were three times more likely to exhibit depression symptoms (OR = 3.125, 95% CI = 1.294–7.549) and four times more likely to exhibit anxiety symptoms (OR = 4.489, 95% CI = 1.812–11.121) (Table 3 and Table 4).

### 3.3. Levels of Social Support

The Mann-Whitney U test was employed to compare the means of social support scores between participants exhibiting symptoms of anxiety and depression and those without clinically significant symptoms. The Spearman’s rho test was used to examine how social support correlated with symptoms of depression and anxiety.

The Spearman’s rho test was used to assess how social support differs with symptoms of depression and anxiety. Among participants, 50.3% scored a 9 out of 9 in the appraisal section, while 65.3% scored a 6 out of 6 in the tangible section of the ISEL-12. Additionally, 66.7% of participants scored 6 out of 9 in the belonging section (Table 5). A higher score indicates a higher level of support. These scores did not conform to a normal distribution. Most participants scored 21 (38.1%, *n* = 168) on social support, with the next highest score being 18 (14.3%, *n* = 168).

When evaluating social support alongside depression and anxiety, the obtained correlation coefficients were −0.137 and −0.150, with *p*-values of 0.076 and 0.052 (both > 0.05), respectively (Table 6 and Table 7). This indicates that a low, negative correlation exists between these variables, although not significant. When assessing the sub-scales of social support individually with depression and anxiety, higher scores in belonging and tangible support significantly correlated with less symptoms of depression (rho = −0.164 and rho = −0.158, respectively), as reflected by lower scores on the PHQ-8 (*p* = 0.034 and 0.040, respectively) (Table 6). Conversely, higher scores in appraisal support were significantly correlated with fewer symptoms of anxiety (rho = −0.161), as indicated by lower scores on the GAD-7 (*p* = 0.037) (Table 7).

The mean rank scores of the participants without depression symptoms were significantly different from the mean rank scores of the participants with depression symptoms in tangible (89.02 vs. 73.29, *p* = 0.040) and belonging support (88.04 vs. 71.97, *p* = 0.034). When comparing the participants without anxiety symptoms with the participants with anxiety symptoms, their mean ranks were significantly different in appraisal support (88.25 vs. 68.64, *p* = 0.037) (Table 6 and Table 7).

The findings observed in the bivariate analyses were further analyzed using binary logistic regression models. The results show that perceived income was the only factor significantly associated with symptoms of depression (OR = 0.425; *p* = 0.031) and anxiety (OR = 0.292; *p* = 0.011) These values indicate that a better perception of income is related to a lower likelihood of experiencing these symptoms (OR < 1) (Table 8).

## 4. Discussion

### 4.1. Discussion of Results

The purpose of this secondary analysis was to determine whether sociodemographic factors, financial burden, and social support influenced the prevalence of psychological distress among our sample of 208 women with breast cancer in the southern region of PR. Symptoms of depression and anxiety were the metrics used to assess psychological distress in the participants. Knowing if social determinants affect how patients manage their disease and if so, which determinants and in which way they impact the patient, can provide more evidence to incorporate other aspects like cost, mental health care, coping mechanisms, and support from the government and social network as part of the treatment plan.

The analysis performed in this study revealed that older participants are associated with having lower symptoms of depression and anxiety, while younger participants between the ages of 50 and 64 are associated with higher symptoms of depression. One possible explanation for this finding is that people over 65 tend to have less financial toxicity than those ages 50–64 (Knight et al., 2018). Older participants at retirement age were more likely to feel that their income was sufficient to cover all expenses. Additionally, lower perceived social support and a sense of vulnerability among older patients can lead to more discussions about costs with their doctors, along with spending more time on these conversations. Discussing treatment costs between a patient and their doctor can help reduce financial toxicity by considering the patient’s needs and expenses when making medical decisions (Hamel et al., 2017).

A study in Spain found that women with breast cancer, particularly those aged 50 to 65, were more likely to experience higher levels of depressive symptoms compared to both younger and older women (Domènech-Abella et al., 2017; Puigpinós-Riera et al., 2018), which aligns with the findings of this analysis. However, other studies contrast with the results of this analysis, where older women with breast cancer were associated with a higher risk of depression in Greece, while younger women with breast cancer were less likely to experience depression compared to older women in Lithuania (Bulotiene et al., 2008; NCI, n.d.a). Another study showed that age is also a predicting factor for resilience, with higher levels of resilience more likely seen in older women with breast cancer. They found a correlation between resilience and levels of anxiety and depression. Levels of resilience presented a negative relationship with depression symptoms and a positive relationship with anxiety symptoms (Fradelos et al., 2017).

No significant association was found between annual household income and the presence of anxiety or depression symptoms. However, when considering whether their income was sufficient to cover all needs and expenses, a significant association emerged with lower levels of anxiety and depression symptoms. The amount of expenses and how participants managed their income to meet financial obligations had a greater influence on their perceived financial toxicity than their actual earnings. These financial responsibilities are more prevalent among women aged 50 to 64 than in those over 65 (Knight et al., 2018). Other studies that measure financial burden show associations with mental health, as well as with physical health. A study that objectively defined a high financial burden as “a ratio of annual family out-of-pocket health care expenditures to family income higher than 10% or 20%” found that financial burden was associated with lower quality of life, physical health, and nonspecific psychological distress in patients with cancer. However, they didn’t find a significant association between financial burden and depressed mood. A study that used subjective questions to establish financial burden, like reported worrying or inability to cover medical costs, borrowing money/declared bankruptcy, or other financial sacrifices, reported a stronger association with mental health. Cancer patients with higher financial burden were 1.95 times more likely to exhibit depressed mood and 3.54 times more likely to worry about cancer recurrence (Kale & Carroll, 2016; Park & Look, 2018).

No significant association was found between employment status, education, or type of health insurance and depression or anxiety. However, other studies have indicated that lower income, education level, and social class significantly influence the levels of anxiety and depression in various regions of the world (Puigpinós-Riera et al., 2018; Tsaras et al., 2018). Others have shown that a medium income is associated with a higher risk of depression, followed by low income and high income (Domènech-Abella et al., 2017).

Marital status has also been shown to be associated with mood and characteristics of anxiety and depression, with widowed women with breast cancer exhibiting higher symptoms, followed by divorced women (Bulotiene et al., 2008; Shrestha et al., 2017; Tsaras et al., 2018). Patients who are not married also present a higher association with financial toxicity (Knight et al., 2018). Additionally, women in joint families were associated with having a better mood than those with a nuclear family (Shrestha et al., 2017). Joint families consist of more than the parents and their children, which provides a more supportive network. However, in this study, no significant association was found in either anxiety or depression according to marital status or living situation. Some studies even suggest that living alone leads to lower levels of anxiety and depression (Puigpinós-Riera et al., 2018). Support from partners, family, and friends is considered a protective factor against depression, which means that the nature and quality of these relationships have an influence on the risk of depression (Gariépy et al., 2016).

This analysis showed that higher levels of social support were associated with lower levels of anxiety and depression. This compares with other studies that assess social support, where factors like strong family relationships and functioning were associated with lower anxiety and depression, while social isolation was associated with higher depression (Civilotti et al., 2021; Puigpinós-Riera et al., 2018). Some studies illustrate that the quality of the network and frequency of social contact are more important than the size of the network (Domènech-Abella et al., 2017). Others illustrate that the number of people in your network, the amount of perceived emotional and tangible support, and the number of social activities are associated with lower depressive symptoms (Falcón et al., 2009). Social support can come in many ways, like having a good family relationship, connection with friends, participating in groups with similar hobbies, beliefs, and aspirations, and having people you can trust and feel comfortable with. Specifically, support with practical assistance and material resources, as well as being connected with people in their lives, were associated with lower levels of depression. The presence of emotionally nurturing interactions was associated with lower levels of anxiety.

Additionally, participants with no depression or anxiety symptoms had higher mean rank scores in all types of social support than those with depression or anxiety symptoms. However, participants with no depression symptoms had a higher statistically significant mean rank score in tangible and belonging support than those with depression symptoms, while participants with no anxiety had a higher statistically significant mean rank score in appraisal support than those with anxiety symptoms.

Since PR is an island prone to natural disasters such as hurricanes and earthquakes, its population is even more vulnerable to psychological distress. In recent years, Hurricane Irma and Hurricane Maria (2017) and Hurricane Fiona (2019) left millions of islanders without power, clean water, food, telecommunications, and access to roads. Additionally, the earthquakes that particularly impacted the southern coast of the island in 2019–2020, followed by the COVID-19 pandemic, further exacerbated the stress experienced by the Puerto Rican population. PTSD following trauma exposure often occurs alongside other comorbidities, such as major depressive disorder and generalized anxiety disorder (Brown et al., 2020; Fortin et al., 2021; Marino et al., 2022; Peña-Vargas et al., 2022).

However, a study found that people living in PR after Hurricane Maria in 2017 experienced lower levels of depression. This population also demonstrates higher levels of social support compared to Puerto Ricans before Hurricane Maria. The reduction in depression may be attributed to the increase in social support following the natural disaster, which acts as a protective factor in how people cope with their problems, even when they are more vulnerable to psychological distress (Mattei et al., 2022).

In PR, social connections are vital for resilience in adverse situations, such as being diagnosed with cancer. Participants in this study, conducted between 2018 and 2023, demonstrated an overall high level of social support. When compared to the U.S. population, individuals living in PR exhibit higher levels of depression, despite having greater social support than mainland residents, including Puerto Ricans. Additionally, Puerto Ricans face higher rates of poverty and unemployment (Canino et al., 2019; Cohen et al., 1985). When adjusting for social support among Puerto Ricans on the mainland, the risk of anxiety and depression was no longer significant, but the risk of depression remained higher than that of the US population. However, islanders exhibited lower levels of anxiety (Falcón et al., 2009).

When psychosocial and sociodemographic variables were considered simultaneously, income perception remained the main predictor of psychological symptoms. These findings reaffirm what previous studies have already indicated: psychological distress is more strongly influenced by how people perceive their economic problems than by their objective income level. This remained even when considering other relevant structural factors (age, educational level, marital status, employment, type of health insurance, or depression) that affect the emotional well-being of women with breast cancer in Puerto Rico. A study conducted in Ireland reported that financial stress derived from the diagnosis was associated with a nearly threefold increase in the risk of depression and anxiety, even when adjusting for clinical and sociodemographic variables (Sharp et al., 2013). As in their study, our sample highlights the importance of focusing on subjective economic conditions to understand the emotional well-being of cancer patients. Although the other variables did not show statistically significant associations, their inclusion in the model allowed for controlling for potential confounders, which strengthens the internal validity of the test by revealing more precise associations.

### 4.2. Findings Implications

The findings of this study provide crucial insight into what are the specific needs of the communities evaluated and therefore, prepare community-based interventions to address gaps in patient care. It informs ongoing efforts like “Mental Health CPR: Transforming Cancer Survivors’ Mental Health with Community Participatory Reach” (Protocol ID# 2303136895), also led by Castro-Figueroa, E.M., Armaiz, G.N., and colleagues at Ponce Health Sciences University/Ponce Research Institute (PRI). This initiative is designed to promote primary mental health prevention strategies by “reducing stress, promoting mental health, managing emotional distress, and supporting overall well-being” in cancer patients in the southern region of Puerto Rico.

Efforts are needed to prevent socioeconomic inequalities from further hindering patient treatment and exacerbating the prevalence of comorbidities in these populations. These issues must be addressed from a multidisciplinary perspective, involving family and friends, the community, government agencies, social workers, and healthcare professionals, including mental health providers. When treating a patient with cancer, it is essential to consider these factors and others that can impact healthcare delivery and outcomes. Psychosocial interventions can also be implemented to help these women and provide them emotional support within their family and community, including support groups specific for women with breast cancer, offering a sense of belonging and adequacy in what they are experiencing.

## 5. Future Directions

The findings reported in this study support further exploration of variables that can influence the perception of financial well-being, particularly those included in the COST-FACIT questionnaire: loss of income, reliance on savings, treatment costs, and financial responsibilities. Future studies could also delve into aspects such as food and housing insecurity, dependents, transportation access, health and financial literacy, geographical location, and the availability and awareness of resources within their community.

## 6. Conclusions

### Study Strengths and Limitations

Patients with cancer encounter various challenges during their recovery process, beyond just debilitating physical ailments. Their experience can be influenced by factors such as social support and financial stressors. This study emphasizes the importance of social connections and tangible support in the resilience of women with BC in Puerto Rico. It also highlights how the perception of financial burden is significantly associated with the mental health and well-being of this population. Among the strengths of this study is the inclusion of a significant sample of women with breast cancer in southern Puerto Rico, which allows the results to be relevant to a population that has been underrepresented in the scientific literature. Additionally, validated instruments (PHQ-8 and GAD-7) were used to measure psychological symptoms. The use of logistic regression models, adjusting for relevant variables and controlling for potential confounders, strengthens the study’s internal validity.

However, it is acknowledged that the study had several limitations that should be considered when interpreting the results. First, the use of self-reported measures, which may be subject to recall bias, is noted. Furthermore, although the cohort design offers the advantage of assessing the outcome over time, definitive causality between the measured variables could not be established. Generalizability of the study results is limited to the southern region of Puerto Rico and not to other populations in the geographic context. Even though this study evaluates several social determinants of health (age, education level, employment status, living situation, marital status, and type of health insurance), it fails to evaluate other important social determinants that could also prove to influence the mental health outcomes of BC patients. These should be incorporated in future research to offer a more comprehensive understanding of what affects the patient and their treatment, and incorporate this information into patient care.

## Figures and Tables

**Table 1:** Sociodemographic Characteristics.

Characteristics	*n*	%	Characteristics	*n*	%
**Age (years)**	***n* = 208**		**Metastasis**	***n* = 174**	
20–34	7	(3.4%)	Yes	26	(14.9%)
35–49	58	(27.9%)	No	148	(85.1%)
50–64	75	(36.1%)	**Annual household income**	***n* = 204**	
65–79	64	(30.8%)	Less than $12,000	84	(41.2%)
80 or over	4	(1.9%)	$12,001–$19,000	43	(21.1%)
**Marital status**	***n* = 208**		$19,001–$35,000	49	(24%)
Single, never married	41	(19.7%)	$35,001–$60,000	24	(11.8%)
Married	92	(44.2%)	$60,001–$100,000	3	(1.5%)
Consensual union	15	(7.2%)	$100,001–$250,000	1	(0.5%)
Divorced/Separated	30	(14.4%)	**Is your income enough?**	***n* = 206**	
Widow	30	(14.4%)	No	123	(59.7%)
**Living with:**	*n* = 208 *		Yes	83	(40.3%)
Spouse/partner	106	(51%)	**Education level**	***n* = 208**	
Parents	12	(5.8%)	No elementary school	2	(1%)
Other family members	12	(5.8%)	Elementary school	6	(2.9%)
Alone	32	(15.4%)	Middle school	14	(6.7%)
Young children	48	(23.1%)	High school	55	(26.4%)
Older offspring	51	(24.5%)	Technical/Associate’s	47	(22.6%)
Other	6	(2.9%)	Bachelor’s	61	(29.3%)
**Family history of cancer**	***n* = 157**		Master’s or Doctorate	23	(11.1%)
Yes	134	(85.4%)	**Health insurance**	***n* = 205**	
No	23	(14.6%)	Medicare	22	(10.7%)
**Family history of mental health**	***n* = 199**		Private	69	(33.7%)
Yes	89	(44.7%)	Government insurance	101	(49.3%)
No	110	(55.3%)	None	1	(0.5%)
**Depression**	***n* = 175**		Both (private and gov.)	10	(4.9%)
Yes	24	(13.7%)	Other	2	(1%)
No	151	(86.3%)	**Employment status**	***n* = 208**	
**Taking SSRIs**	***n* = 207**		Full time	53	(25.5%)
Yes	26	(12.6%)	Part time	14	(6.7%)
No	181	(87.4%)	Seasonal	2	(1%)
**Treatment**	*n* = 208 *		Retired	50	(24%)
Chemotherapy	117	(56.3%)	Unemployed	47	(22.6%)
Hormone therapy	67	(32.2%)	Disabled	22	(10.6%)
Radiotherapy	98	(47.1%)	Other	20	(9.6%)
Surgery	134	(64.4%)			

SSRIs, Selective Serotonin Reuptake Inhibitors (Citalopram, Escitalopram, Fluoxetine, Paroxetine, Sertraline, Vilazodone); *n* = number of participants that answered the question; * participants were allowed to have multiple choice answers.

**Table 2:** Depression and Anxiety Symptoms.

Scale	*n*	%	Scale	*n*	%
**PHQ-8**	***n* = 208**		**GAD-7**	***n* = 208**	
0–4 (minimal)	124	(59.6%)	0–4 (minimal)	132	(63.5%)
5–9 (mild)	44	(21.2%)	5–9 (mild)	45	(21.6%)
10–14 (moderate)	25	(12.0%)	10–14 (moderate)	22	(10.6%)
15–19 (moderately severe)	14	(6.7%)	15–21 (severe)	9	(4.3%)
20–24 (severe)	1	(0.5%)			

PHQ-8, Patient Health Questionnaire Depression Scale; GAD-7, Generalized Anxiety Disorder Questionnaire.

**Table 3:** Association between Characteristics and Depression in Breast Cancer Patients in PR.

	No Depression		Depression		Increased Depression	
Characteristics	*n*	%	*n*	%	OR (95% Cl)	*p*-Value
**Age**						
Less than 50	52	(80%)	13	(20%)	1.065 (0.509–2.228)	0.868
50–64	55	(73.3%)	20	(26.7%)	2.036 (1.012–4.096)	0.044 *
65 or more	60	(89.6%)	7	(10.4%)	0.378 (0.158–0.907)	0.025 *
**Income**						
Less than $12,000	69	(82.1%)	15	(17.9%)	0.873 (0.427–1.787)	0.711
$12,001–$19,000	35	(81.4%)	8	(18.6%)	0.944 (0.398–2.236)	0.895
$19,001–$35,000	41	(83.7%)	8	(16.3%)	0.794 (0.337–1.866)	0.596
$35,001–$60,000	16	(66.7%)	8	(33.3%)	2.371 (0.933–6.027)	0.070
$60,001–$100,000	3	(100%)	0	(0%)	-	0.393
$100,001–$250,000	1	(100%)	0	(0%)	-	0.625
**Is your income enough?**						
Yes	73	(89%)	9	(11%)	0.382 (0.171–0.855)	0.017 *
No	93	(75.6%)	30	(24.4%)		
**Living Situation**						
Spouse/partner	83	(79%)	22	(21%)	1.237 (0.619–2.473)	0.547
Parents	10	(83.3%)	2	(16.7%)	0.826 (0.174–3.928)	1.000
Other family members	12	(100%)	0	(0%)	-	0.128
Young children	39	(81.3%)	9	(18.8%)	0.953 (0.418–2.172)	0.909
Older offspring	39	(76.5%)	12	(23.5%)	1.407 (0.654–3.024)	0.381
Alone	28	(87.5%)	4	(12.5%)	0.552 (0.182–1.674)	0.341
**Employment**						
Employed	51	(77.3%)	15	(22.7%)	1.365 (0.664–2.803)	0.396
Unemployed	34	(73.9%)	12	(26.1%)	1.676 (0.773–3.636)	0.188
Retired	44	(88%)	6	(12%)	0.493 (0.194–1.255)	0.132
Disabled	16	(72.7%)	6	(27.3%)	1.665 (0.607–4.570)	0.318
**Education level**						
No Degree	16	(72.7%)	6	(27.3%)	1.665 (0.607–4.570)	0.318
High School Degree	45	(83.3%)	9	(16.7%)	0.787 (0.348–1.782)	0.565
Higher Degree	106	(80.9%)	25	(19.1%)	0.959 (0.470–1.958)	0.909
**Marital Status**						
Married	73	(80.2%)	18	(19.8%)	1.054 (0.526–2.109)	0.883
Consensual Union	10	(66.7%)	5	(33.3%)	2.243 (0.721–6.973)	0.154
Single	34	(82.9%)	7	(17.1%)	0.830 (0.338–2.038)	0.684
Divorced	23	(76.7%)	7	(23.3%)	1.328 (0.526–3.335)	0.547
Widowed	27	(90%)	3	(10%)	0.420 (0.121–1.462)	0.214
**Health Insurance**						
Private	59	(85.5%)	10	(14.5%)	0.620 (0.282–1.360)	0.230
Medicare	20	(91%)	2	(9%)	0.392 (0.088–1.752)	0.262
Government	76	(76%)	24	(24%)	1.874 (0.917–3.827)	0.082
Private and Gov.	9	(90%)	1	(10%)	0.456 (0.056–3.711)	0.691
**Taking SSRIs**	16	(61.5%)	10	(38.5%)	3.125 (1.294–7.549)	0.009 *
**Depression**	13	(54.2%)	11	(45.8%)	3.855 (1.560–9.524)	0.002 *

* *p*-value < 0.05, statistically significant; Odds Ratio (OR), the odds of having the disease in exposed vs. unexposed groups; Level of Confidence Interval (95% CI); Employed, full-time and part-time; when *n* < 5, Fisher’s exact test was used.

**Table 4:** Association between Characteristics and Anxiety in Breast Cancer Patients in PR.

	No Anxiety		Anxiety		Increased Anxiety	
Characteristics	*n*	%	*n*	%	OR (95%, Cl)	*p*-Value
**Age**						
Less than 50	52	(80%)	13	(20%)	1.618 (0.745–3.518)	0.221
50–64	59	(78.7%)	16	(21.3%)	1.966 (0.919–4.206)	0.078
65 or more	64	(95.5%)	3	(4.5%)	0.179 (0.053–0.613)	0.002 *
**Income**						
Less than $12,000	74	(88.1%)	10	(11.9%)	0.639 (0.284–1.439)	0.280
$12,001–$19,000	36	(83.7%)	7	(16.3%)	1.094 (0.436–2.741)	0.848
$19,001–$35,000	40	(81.6%)	9	(18.4%)	1.385 (0.590–3.252)	0.455
$35,001–$60,000	19	(79.2%)	5	(20.8%)	1.538 (0.528–4.482)	0.430
$60,001–$100,000	3	(100%)	0	(0%)	-	0.457
$100,001–$250,000	1	(100%)	0	(0%)	-	0.669
**Is your income enough?**						
Yes	75	(91.5%)	7	(8.5%)	0.366 (0.150–0.891)	0.023 *
No	98	(79.7%)	25	(20.3%)		
**Living Situation**						
Spouse/partner	87	(82.9%)	18	(17.1%)	1.300 (0.609–2.777)	0.497
Parents	11	(91.7%)	1	(83.3%)	0.481 (0.060–3.860)	0.697
Other family members	12	(100%)	0	(0%)	-	0.220
Young children	40	(83.3%)	8	(16.7%)	1.125 (0.469–2.697)	0.792
Older offspring	41	(80.3%)	10	(19.6%)	1.486 (0.651–3.391)	0.345
Alone	28	(87.5%)	4	(12.5%)	0.750 (0.244–2.305)	0.792
**Employment**						
Employed	53	(80.3%)	13	(19.7%)	1.575 (0.735–3.421)	0.249
Unemployed	40	(87%)	6	(13%)	0.779 (0.300–2.024)	0.607
Retired	44	(88%)	6	(12%)	0.687 (0.265–1.779)	0.437
Disabled	17	(77.3%)	5	(22.7%)	1.721 (0.586–5.055)	0.319
**Education level**						
No Degree	21	(95.5%)	1	(4.5%)	0.237 (0.031–1.824)	0.211
High School Degree	46	(85.2%)	8	(14.8%)	0.935 (0.392–2.227)	0.879
Higher Degree	108	(83.1%)	22	(16.9%)	1.585 (0.692–3.631)	0.273
**Marital Status**						
Married	78	(85.7%)	13	(14.3%)	0.851 (0.396–1.830)	0.679
Consensual Union	10	(66.7%)	5	(33.3%)	3.056 (0.969–9.631)	0.047 *
Single	36	(87.8%)	5	(12.2%)	0.715 (0.257–1.987)	0.519
Divorced	24	(80%)	6	(20%)	1.452 (0.541–3.894)	0.457
Widowed	27	(90%)	3	(10%)	0.567 (0.161–1.994)	0.584
**Health Insurance**						
Private	58	(84.1%)	11	(15.9%)	1.030 (0.465–2.280)	0.943
Medicare	19	(86.4%)	3	(13.6%)	0.830 (0.231–2.998)	1.000
Government	83	(83%)	17	(17%)	1.215 (0.571–2.588)	0.613
Private and Gov.	10	(100%)	0	(0%)	-	0.368
**Taking SSRIs**	16	(61.5%)	10	(38.5%)	4.489 (1.812–11.121)	<0.001 *
**Depression**	15	(62.5%)	9	(37.5%)	3.900 (1.507–10.095)	0.003 *

* *p*-value < 0.05, statistically significant; Odds Ratio (OR), the odds of having the disease in exposed vs. unexposed groups; Level of Confidence Interval (95% CI); Employed, full-time and part-time; when *n* < 5, Fisher’s exact test was used.

**Table 5:** Social Support (ISEL-12 scores) in Women with Breast Cancer in PR.

Scores	Belonging Support (0–9)		Tangible Support (0–6)		Appraisal Support (0–9)	
	Frequency	Percent	Frequency	Percent	Frequency	Percent
0.00	2	1.2%	2	1.2%	5	3%
1.00	0	0%	4	2.4%	2	1.2%
2.00	4	2.4%	2	1.2%	1	0.6%
3.00	5	3.0%	29	17.1%	10	5.9%
4.00	5	3.0%	8	4.7%	3	1.8%
5.00	14	8.3%	14	8.2%	3	1.8%
6.00	112	66.7%	111	65.3%	29	17.2%
7.00	9	5.4%	-	-	10	5.9%
8.00	6	3.6%	-	-	21	12.4%
9.00	11	6.5%	-	-	85	50.3%
Total (*n*)	168	100.0%	170	100.0%	169	100.0%

**Table 6:** Social Characteristics and Depression in Women with Breast Cancer in PR.

Categories	No Depression	Depression	*p*-Value	Correlation Coefficient (rho)	*p*-Value
	Mean Rank	Mean Rank			
Social support	87.93	72.36	0.076	−0.137	0.076
Tangible	89.02	73.29	0.040 *	−0.158	0.040 *
Appraisal	87.91	74.64	0.117	−0.121	0.117
Belonging	88.04	71.97	0.034 *	−0.164	0.034 *

Mean Rank, Mann-Whitney U test; Correlation Coefficient, Spearman’s rho test; * *p*-value < 0.05.

**Table 7:** Social characteristics and anxiety in women with breast cancer in PR.

Categories	No Anxiety	Anxiety	*p*-Value	Correlation Coefficient (rho)	*p*-Value
	Mean Rank	Mean Rank			
Social support	87.65	68.73	0.053	−0.150	0.052
Tangible	88.01	73.28	0.083	−0.134	0.083
Appraisal	88.25	68.64	0.037 *	−0.161	0.037 *
Belonging	86.20	76.02	0.228	−0.093	0.229

Mean Rank, Mann-Whitney U test; Correlation Coefficient, Spearman’s rho test; * *p*-value < 0.05.

**Table 8:** Factors associated with symptoms of depression (PHQ-8) and anxiety (GAD-7) in women with breast cancer in Puerto Rico.

Variables	Outcome	Sig. (*p*-Value)	OR (95%, CI)
**Depression (PHQ-8)**			
Age	Depression (PHQ-8)	0.762	0.995 (0.961–1.029)
Last Academic Level	Depression (PHQ-8)	0.648	0.931 (0.685–1.265)
Marital Status	Depression (PHQ-8)	0.348	0.894 (0.707–1.130)
Income Perception	Depression (PHQ-8)	0.031 *	0.425 (0.195–0.925)
Annual Income	Depression (PHQ-8)	0.727	1.067 (0.749–1.532)
Employment	Depression (PHQ-8)	0.527	1.267 (0.609–2.636)
Type of Insurance	Depression (PHQ-8)	0.172	1.216 (0.918–1.609)
Depression	Depression (PHQ-8)	0.138	2.125 (0.785–5.755)
**Anxiety (GAD-7)**			
Age	Anxiety (GAD-7)	0.272	0.981 (0.949–1.015)
Last Academic Level	Anxiety (GAD-7)	0.094	1.326 (0.953–1.845)
Marital Status	Anxiety (GAD-7)	0.699	1.057 (0.798–1.401)
Income Perception	Anxiety (GAD-7)	0.011 *	0.292 (0.113–0.754)
Annual Income	Anxiety (GAD-7)	0.608	1.101 (0.763–1.589)
Employment	Anxiety (GAD-7)	0.800	1.125 (0.452–2.803)
Type of Insurance	Anxiety (GAD-7)	0.463	0.877 (0.619–1.245)
Depression	Anxiety (GAD-7)	0.821	1.150 (0.342–3.873)

Logistic Regression; * *p*-value < 0.05.

## Data Availability

The data used in this study were obtained from a previously collected dataset and are not publicly available due to privacy and ethical restrictions. Requests to access the dataset should be directed to the corresponding author and will be evaluated in accordance with institutional policies and IRB approval.

## Abbreviations

The following abbreviations are used in this manuscript:

ACS	American Cancer Society
BC	Breast Cancer
CI	Confidence Interval
DSM-V	Diagnostic and Statistical Manual of Mental Dis-orders
GAD-7	Generalized Anxiety Disorder
ISEL-12	Interpersonal Support Evaluation List
MDPI	Multidisciplinary Digital Publishing Institute
n	sample
NCI	National Cancer Institute
OR	Odds Ratio
p	p value
PHQ-8	Patient Health Questionnaire
PR	Puerto Rico
PTSD	Post-traumatic stress disorder
SSRIs	Selective Serotonin Reuptake Inhibitors

## References

[B1-behavsci-15-00915] American Cancer Society (2022). Breast cancer facts & figures 2022–2024.

[B2-behavsci-15-00915] American Cancer Society (2024). Cancer facts & figures for hispanic/latino people 2024–2026.

[B3-behavsci-15-00915] American Psychiatric Association, DSM-5 Task Force (2013). Diagnostic and statistical manual of mental disorders: DSM-5.

[B4-behavsci-15-00915] Bolton D. (2023). A revitalized biopsychosocial model: Core theory, research paradigms, and clinical implications. Psychological Medicine.

[B5-behavsci-15-00915] Breidenbach C., Heidkamp P., Hiltrop K., Pfaff H., Enders A., Ernstmann N., Kowalski C. (2022). Prevalence and determinants of anxiety and depression in long-term breast cancer survivors. BMC Psychiatry.

[B6-behavsci-15-00915] Brown L. C., Murphy A. R., Lalonde C. S., Subhedar P. D., Miller A. H., Stevens J. S. (2020). Posttraumatic stress disorder and breast cancer: Risk factors and the role of inflammation and endocrine function. Cancer.

[B7-behavsci-15-00915] Bulotiene G., Veseliunas J., Ostapenko V., Furmonavicius T. (2008). Women with breast cancer: Relationships between social factors involving anxiety and depression. Archives of Psychiatry & Psychotherapy.

[B8-behavsci-15-00915] Caceres M. C., Nadal-Delgado M., Lopez-Jurado C., Perez-Civantos D., Guerrero-Martin J., Duran-Gomez N. (2022). Factors related to anxiety, depressive symptoms and quality of life in breast cancer. International Journal of Environmental Research and Public Health.

[B9-behavsci-15-00915] Canino G., Shrout P. E., NeMoyer A., Vila D., Santiago K. M., Garcia P., Quiñones A., Cruz V., Alegria M. (2019). A comparison of the prevalence of psychiatric disorders in Puerto Rico with the United States and the Puerto Rican population of the United States. Social Psychiatry and Psychiatric Epidemiology.

[B10-behavsci-15-00915] Castro-Figueroa E. M., Acevedo K. I., Peña-Vargas C. I., Torres-Blasco N., Flores I., Colón-Echevarria C. B., Maldonado L., Rodríguez Z., Aquino-Acevedo A. N., Jim H., Lazaro M. I., Armaiz-Peña G. N. (2021). Depression, anxiety, and social environmental adversity as potential modulators of the immune tumor microenvironment in breast cancer patients. Medical Sciences.

[B11-behavsci-15-00915] Civilotti C., Botto R., Maran D. A., Leonardis B. D., Bianciotto B., Stanizzo M. R. (2021). Anxiety and depression in women newly diagnosed with breast cancer and waiting for surgery: Prevalence and associations with socio-demographic variables. Medicina.

[B12-behavsci-15-00915] Cohen S., Mermelstein R., Kamarck T., Hoberman H. M. (1985). Measuring the functional components of social support. Social support: Theory, research and applications.

[B13-behavsci-15-00915] de Sousa Barros A. E., Conde C. R., Lemos T. M. R., Kunz J. A., da Silva Marques M. D. L. (2018). Feelings experienced by women when receiving the diagnosis of breast cancer. Journal of Nursing.

[B14-behavsci-15-00915] Domènech-Abella J., Lara E., Rubio-Valera M., Olaya B., Moneta M. V., Rico-Uribe L. A., Ayuso-Mateos J. L., Mundó J., Haro J. M. (2017). Loneliness and depression in the elderly: The role of social network. Social Psychiatry and Psychiatric Epidemiology.

[B15-behavsci-15-00915] Engel G. L. (1977). The need for a new medical model: A challenge for biomedicine. Science.

[B16-behavsci-15-00915] Falcón L. M., Todorova I., Tucker K. (2009). Social support, life events, and psychological distress among the Puerto Rican population in the Boston area of the United States. Aging & Mental Health.

[B17-behavsci-15-00915] Fenech B., Gaffiero D. (2025). Investigating barriers and facilitators to engagement with the national breast screening programme among women in Malta: A systematic review. Advances in Public Health.

[B18-behavsci-15-00915] Fortin J., Leblanc M., Elgbeili G., Cordova M. J., Marin M. F., Brunet A. (2021). The mental health impacts of receiving a breast cancer diagnosis: A meta-analysis. British Journal of Cancer.

[B19-behavsci-15-00915] Fradelos E. C., Papathanasiou I. V., Veneti A., Daglas A., Christodoulou E., Zyga S., Kourakos M. (2017). Psychological distress and resilience in women diagnosed with breast cancer in Greece. Asian Pacific Journal of Cancer Prevention.

[B20-behavsci-15-00915] Gariépy G., Honkaniemi H., Quesnel-Vallée A. (2016). Social support and protection from depression: Systematic review of current findings in Western countries. British Journal of Psychiatry.

[B21-behavsci-15-00915] Hamel L. M., Penner L. A., Eggly S., Chapman R., Klamerus J. F., Simon M. S., Stanton S. C., Albrecht T. L. (2017). Do patients and oncologists discuss the cost of cancer treatment? An observational study of clinical interactions between African American patients and their oncologists. Journal of Oncology Practice.

[B22-behavsci-15-00915] Islami F., Bispo J. B., Lee H., Wiese D., Yabroff K. R., Bandi P., Sloan K., Patel A. V., Daniels E. C., Kamal A. H., Guerra C. E., Dahut W. L., Jemal A. (2024). American Cancer Society’s report on the status of cancer disparities in the United States, 2023. CA Cancer Journal for Clinicians.

[B23-behavsci-15-00915] Kale H. P., Carroll N. V. (2016). Self-reported financial burden of cancer care and its effect on physical and mental health-related quality of life among US cancer survivors. Cancer.

[B24-behavsci-15-00915] Khazi F., Angolkar M., Bhise R., Ahmed I. (2023). Psychosocial impact at diagnosis and coping strategies among women with breast cancer-A qualitative study. Clinical Epidemiology and Global Health.

[B25-behavsci-15-00915] Knight T. G., Deal A. M., Dusetzina S. B., Muss H. B., Choi S. K., Bensen J. T., William G. R. (2018). Financial toxicity in adults with cancer: Adverse outcomes and noncompliance. Journal of Oncology Practice.

[B26-behavsci-15-00915] Kroenke K., Strine T. W., Spitzer R. L., Williams J. B., Berry J. T., Mokdad A. H. (2009). The PHQ-8 as a measure of current depression in the general population. Journal of Affective Disorders.

[B27-behavsci-15-00915] Liu Y., Cao C. (2014). The relationship between family history of cancer, coping style and psychological distress. Pakistan Journal of Medical Sciences.

[B28-behavsci-15-00915] Lueboonthavatchai P. (2007). Prevalence and psychosocial factors of anxiety and depression in breast cancer patients. Journal-Medical Association of Thailand.

[B29-behavsci-15-00915] Marino P., Touzani R., Pakradouni J., Ben Soussan P., Gravis G. (2022). The psychological distress of cancer patients following the COVID-19 pandemic first lockdown: Results from a large French survey. Cancers.

[B30-behavsci-15-00915] Mattei J., Tamez M., O’Neill J., Haneuse S., Mendoza S., Orozco J., Lopez-Cepero A., Ríos-Bedoya C. F., Falcón L. M., Tucker K. L., Rodríguez-Orengo J. F. (2022). Chronic diseases and associated risk factors among adults in Puerto Rico after Hurricane Maria. JAMA Network Open.

[B31-behavsci-15-00915] Merz E. L., Roesch S. C., Malcarne V. L., Penedo F. J., Llabre M. M., Weitzman O. B., Navas-Nacher E. L., Perreira K. M., Gonzalez F., Ponguta L. A., Johnson T. P., Gallo L. C. (2014). Validation of interpersonal support evaluation list-12 (ISEL-12) scores among English-and Spanish-speaking Hispanics/Latinos from the HCHS/SOL sociocultural ancillary study. Psychological Assessment.

[B32-behavsci-15-00915] Miles E. (2013). Biopsychosocial model. Encyclopedia of behavioral medicine.

[B33-behavsci-15-00915] National Cancer Institute (n.d.a). SEER cancer stat facts: Female breast cancer.

[B34-behavsci-15-00915] National Cancer Institute (n.d.b). Financial toxicity. NCI dictionary of cancer terms.

[B35-behavsci-15-00915] Park J., Look K. A. (2018). Relationship between objective financial burden and the health-related quality of life and mental health of patients with cancer. Journal of Oncology Practice.

[B36-behavsci-15-00915] Peña-Vargas C., del Río-Rodriguez P., Rosario L. P., Laporte-Estela G., Torres-Blasco N., Rodriguez-Castro Z., Tollinchi-Natali N., Guerrero W. I., Torres P., Armaiz-Pena G. N., Castro-Figueroa E. M. (2025). Losses related to breast cancer diagnosis: The impact on grief and depression symptomatology within the context of Hispanic/Latina patients with breast cancer. Healthcare.

[B37-behavsci-15-00915] Peña-Vargas C., Toro-Morales Y., Valentin P., López M., Rodriguez-Castro Z., Hernandez-Torres R., Tollinchi-Natali N., Torres-Blasco N., Pereira C., Armaiz-Pena G. N., Jim H., Castro-Figueroa E. M. (2022). Impact of seismic activity on access to health care in Hispanic/Latino cancer patients from Puerto Rico. International Journal of Environmental Research and Public Health.

[B38-behavsci-15-00915] Portela M., Sommers B. D. (2015). On the outskirts of national health reform: A comparative assessment of health insurance and access to care in Puerto Rico and the United States. The Milbank Quarterly.

[B39-behavsci-15-00915] Puigpinós-Riera R., Graells-Sans A., Serral G., Continente X., Bargalló X., Domènech M., Espinosa-Bravo M., Grau J., Macia F., Manzanera R., Pla M., Quintana M. J., Sala M., Vidal E. (2018). Anxiety and depression in women with breast cancer: Social and clinical determinants and influence of the social network and social support (DAMA cohort). Cancer Epidemiology.

[B40-behavsci-15-00915] Rodriguez-Rabassa M., Hernández R., Rodriguez Z., Colon-Echevarria C. B., Maldonado L., Tollinchi N., Torres-Marrero E., Mulero A., Albors D., Pérez-Morales J., Flores I., Dutil J., Jim H., Castro E. M., Armaiz-Pena G. N. (2020). Impact of a natural disaster on access to care and biopsychosocial outcomes among Hispanic/Latino cancer survivors. Scientific Reports.

[B41-behavsci-15-00915] Sharp L., Carsin A. E., Timmons A. (2013). Associations between cancer-related financial stress and strain and psychological well-being among individuals living with cancer. Psycho-Oncology.

[B42-behavsci-15-00915] Shrestha J. S., Shrestha A., Sapkota A., Sharma R., Shrestha S., Shestha S., Amayta K. S., Gautam M. (2017). Social support, quality of life and mental health status in breast cancer patients. Cancer Reports and Reviews.

[B43-behavsci-15-00915] Spitzer R. L., Kroenke K., Williams J. B. W., Löwe B. (2006). A brief measure for assessing generalized anxiety disorder: The GAD-7. Archives of Internal Medicine.

[B44-behavsci-15-00915] Torres-Cintrón C. R., Suárez-Ramos T., Román-Ruiz Y., Ortiz-Ortiz K. J., De Jesús-Monge V., Gierbolini-Bermúdez A., Zavala-Zegarra D., Tortolero-Luna G. (2023). Cáncer en Puerto Rico, 2016–2020.

[B45-behavsci-15-00915] Tsaras K., Papathanasiou I. V., Mitsi D., Veneti A., Kelesi M., Zyga S., Fradelos E. C. (2018). Assessment of depression and anxiety in breast cancer patients: Prevalence and associated factors. Asian Pacific Journal of Cancer Prevention.

[B46-behavsci-15-00915] U.S. Census Bureau (2023a). Income in the past 12 months (in 2023 inflation-adjusted dollars)*; American Community Survey, ACS 1-Year Estimates Subject Tables, Table S1901*.

[B47-behavsci-15-00915] U.S. Census Bureau (2023b). American community survey 1-year estimates.

[B48-behavsci-15-00915] Vargas-Ramos C., Colón-Meléndez L., Soldevilla-Irizarry J., Figueroa-Lazú D., Hinojosa J., Bonilla Y. (2023). Pervasive poverty in Puerto Rico: A closer look.

[B49-behavsci-15-00915] Vilela-Estrada A. L., Villarreal-Zegarra D., Toyama M., Carbonel A., Fung C., Carbonetti F. L., Hidalgo-Padilla L., Sureshkumar D. S., Uribe-Restrepo J. M., Olivar N., Gomez-Restrepo C., Brusco L. I., Rodríguez Malagón N., Priebe S., Diez-Canseco F. (2025). Psychometric properties of the patient health questionnaire-8 and general anxiety disorder-7 in adolescents and young adults from three Latin American cities: Internal structure, invariance, internal consistency and divergent validity. Journal of Affective Disorders.

[B50-behavsci-15-00915] Wu X., Wang J., Cofie R., Kaminga A. C., Liu A. (2016). Prevalence of posttraumatic stress disorder among breast cancer patients: A meta-analysis. Iranian Journal of Public Health.

